# Selective and rapid monitoring of dual platelet inhibition by aspirin and P2Y_12 _antagonists by using multiple electrode aggregometry

**DOI:** 10.1186/1477-9560-8-9

**Published:** 2010-05-13

**Authors:** Sandra M Penz, Isabell Bernlochner, Orsolya Tóth, Reinhard Lorenz, Andreas Calatzis, Wolfgang Siess

**Affiliations:** 1 Institute for Prevention of Cardiovascular Diseases, University of Munich, Munich, Germany; 2 Department of Transfusion Medicine and Haemostaseology, University of Munich, Munich, Germany; 3 Current Address: 1st Department of Medicine, University of Pécs, Pécs, Hungary

## Abstract

**Background:**

Poor platelet inhibition by aspirin or clopidogrel has been associated with adverse outcomes in patients with cardiovascular diseases. A reliable and facile assay to measure platelet inhibition after treatment with aspirin and a P2Y_12 _antagonist is lacking. Multiple electrode aggregometry (MEA), which is being increasingly used in clinical studies, is sensitive to platelet inhibition by aspirin and clopidogrel, but a critical evaluation of MEA monitoring of dual anti-platelet therapy with aspirin and P2Y_12 _antagonists is missing.

**Design and Methods:**

By performing *in vitro *and *ex vivo *experiments, we evaluated in healthy subjects the feasibility of using MEA to monitor platelet inhibition of P2Y_12 _antagonists (clopidogrel *in vivo*, cangrelor *in vitro*) and aspirin (100 mg per day *in vivo*, and 1 mM or 5.4 mM *in vitro*) alone, and in combination. Statistical analyses were performed by the Mann-Whitney rank sum test, student' t-test, analysis of variance followed by the Holm-Sidak test, where appropriate.

**Results:**

ADP-induced platelet aggregation in hirudin-anticoagulated blood was inhibited by 99.3 ± 1.4% by *in vitro *addition of cangrelor (100 nM; p < 0.001) and by 64 ± 35% by oral clopidogrel (600 mg) intake (p < 0.05; values are means ± SD). Pre-incubation of blood with aspirin (1 mM) or oral aspirin intake (100 mg/day for 1 week) inhibited arachidonic acid (AA)-stimulated aggregation >95% and 100 ± 3.2%, respectively (p < 0.01). Aspirin did not influence ADP-induced platelet aggregation, either *in vitro *or *ex vivo*. Oral intake of clopidogrel did not significantly reduce AA-induced aggregation, but P2Y_12 _blockade by cangrelor (100 nM) *in vitro *diminished AA-stimulated aggregation by 53 ± 26% (p < 0.01). A feasibility study in healthy volunteers showed that dual anti-platelet drug intake (aspirin and clopidogrel) could be selectively monitored by MEA.

**Conclusions:**

Selective platelet inhibition by aspirin and P2Y_12 _antagonists alone and in combination can be rapidly measured by MEA. We suggest that dual anti-platelet therapy with these two types of anti-platelet drugs can be optimized individually by measuring platelet responsiveness to ADP and AA with MEA before and after drug intake.

## Introduction

Ischemic cardio- and cerebrovascular events remain the leading causes of mortality worldwide, despite the increasing use of angioplasty and pharmacological therapies: According to the 2008 WHO health statistics report [1], deaths from myocardial infarction and stroke are expected to rise from 21.9% of total global mortality in 2004 to 26.3% in 2030. Treatment with aspirin (acetylsalicylic acid, ASA) of patients with cardiovascular disease reduces the risk of ischemic stroke, myocardial infarction, or death by about 25% [2]. Beside ASA, antagonists of the platelet ADP receptor P2Y_12_, such as clopidogrel and recently also prasugrel, have been shown to exert a protective effect in patients with cardio- and cerebrovascular diseases [3-5]. Moreover, dual anti-platelet therapy with ASA and a P2Y_12 _antagonist (clopidogrel or prasugrel) has now been established to prevent thrombotic complications of acute coronary syndromes and percutaneous coronary interventions (PCI) [4-6].

Despite the advances that have been made to date, many studies have shown significant variability of platelet reactivity in patients treated with both ASA and clopidogrel [5,7]. A weak response to ASA [8,9] or clopidogrel [10-14], as determined by *in vitro *platelet function tests, has been significantly associated with adverse outcomes. In this context, there is a need for a reliable assay for platelet function, since many of the platelet function tests used to monitor anti-platelet therapy are not optimal: they show large differences in monitoring anti-platelet drug intake [15]. A recent review concluded that commonly used laboratory tests do not reliably identify nonresponders to anti-platelet therapy [16]. Some researchers regard light transmission aggregometry (LTA) of platelet-rich plasma (PRP) as the gold standard for platelet function testing. LTA is, however, a very poorly standardized technique [17]. LTA variations derive from different procedures for the preparation of PRP, the application of different devices, and different recording times and curve analysis algorithms (final aggregation/maximal aggregation). Additional drawbacks are that LTA is laborious and time consuming and the separation of platelets from other blood cells is not physiological. Moreover, LTA lacks the required sensitivity to determine the effect of P2Y_12 _antagonists. Since signaling via P2Y_1 _induces platelet shape change and reversible aggregation, and signaling through P2Y_12 _amplifies aggregation and renders it irreversible [7], P2Y_12 _receptor blockade only reduces, but does not completely inhibit, ADP-induced aggregation of PRP. Furthermore, LTA also lacks the specificity to measure the effect of P2Y_12 _antagonists in patients concomitantly taking ASA, since ASA can inhibit ADP-induced aggregation of PRP [18].

Dual anti-platelet therapy with ASA plus a P2Y_12 _antagonist (clopidogrel or prasugrel) is increasingly used in patients with acute coronary syndromes undergoing PCI [4-6]. There is a clinical need for a reliable test to measure dual platelet inhibition after ASA and P2Y_12 _antagonist treatment in order to optimize anti-platelet therapy in individual patients. A relatively new system, which is becoming increasingly popular in clinical studies, is multiple electrode aggregometry (MEA) [19]. This method, which is based on impedance aggregometry [20], allows the analysis of platelet aggregation in whole blood. It has been shown that MEA is sensitive to platelet inhibition by ASA [19,21] and clopidogrel [14,21], Recently, high ADP-induced platelet aggregation determined by MEA in patients treated with clopidogrel has been found to be a significant risk factor for stent thrombosis with a hazard ratio of 9.4 [22].

In the present study, we evaluated the use of MEA for monitoring dual anti-platelet therapy with ASA and a P2Y_12 _antagonist. In the first part of the study, we conducted an *in vitro *investigation of important possible confounding factors, such as the influence of P2Y_12 _inhibition with cangrelor on arachidonic acid (AA)-induced aggregation and the effect of ASA on ADP-induced aggregation. In the second part of the investigation, we performed a feasibility study of MEA monitoring of platelet inhibition after oral intake of ASA and clopidogrel, alone and in combination, in healthy volunteers.

## Design and Methods

### Reagents and chemicals

ASA, ADP, apyrase (grade VII), AA and the P2Y_1 _receptor antagonist MRS2179 (*N6*-methyl-2'-deoxyadenosine-3',5'-bisphosphate) were obtained from Sigma (Taufkirchen, Germany). ADPtest, ASPItest (contains AA) and ASA control used in the *ex vivo *study were purchased from Dynabyte (Munich, Germany). Recombinant hirudin (Refludan^®^) was purchased from Schering (Berlin, Germany). The P2Y_12 _receptor antagonist cangrelor (AR-C69931MX;*N6*-2-(methylthioethyl)-2-(3,3,3-trifluoropropylthio)-β, γ-dichloromethylene ATP) was generously provided by Astra-Zeneca R&D Charnwood (Loughborough, UK). Clopidogrel was obtained from Bristol-Myers Squibb Pharma EEIG (Iscover^® ^300 mg, Uxbridge, UK), and ASA from Bayer Vital GmbH (ASA^® ^100 mg, Leverkusen, Germany). All other chemicals were obtained from Merck (Darmstadt, Germany).

### Blood collection and study design

For the *in vitro *experiments, blood samples (10 - 50 ml) were collected following written informed consent from 18 healthy volunteers, who had not taken any anti-platelet drugs for at least 14 days prior to the experiments. The collection of blood samples from healthy volunteers was approved by the Ethics Committee of the University of Munich. The blood samples were drawn by venipuncture using a 19-gauge needle into plastic syringes containing 1/10 volume recombinant hirudin (final concentration in blood: 13 μg/ml) dissolved in 20 mM HEPES, 138 mM NaCl, 1 mM MgCl_2_.6 H_2_O, and 0.36 mM NaH_2_PO_4_, pH 7.4. The first 3 ml of blood were discarded. For the *in-vitro *experiments, different concentrations of cangrelor or MRS2179 were added 1 min before ADP or AA. ASA was dissolved in hirudin-containing buffer solution and drawn together with the anticoagulant into a syringe to obtain a final concentration of 1 mM ASA in the collected blood sample. Platelet aggregation was studied on a Multiplate^® ^analyzer (Dynabyte; Munich, Germany) 45 to 180 min following sampling.

**Figure 1:**
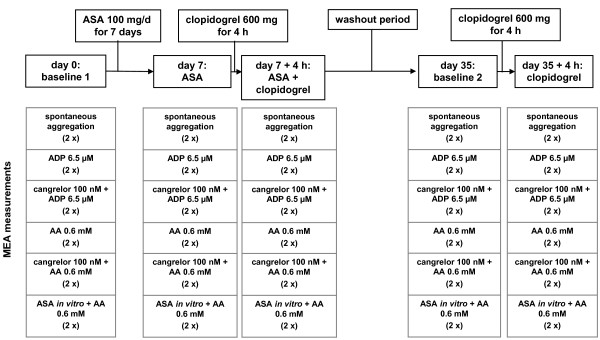
**Design of the *ex vivo *study investigating the effects of oral intake of ASA, clopidogrel or a combination of both drugs on platelet aggregation measured by MEA**.

The *ex vivo *study of the effects of oral intake of ASA, clopidogrel or a combination of the two drugs was carried out by and on five healthy medical doctors, in accordance with the principles of the Helsinki Declaration. All subjects denied taking any medication affecting platelet function in the two weeks prior to this study. Blood samples were collected by venipuncture using a 21-gauge needle into plastic syringes pre-filled with recombinant hirudin (final concentration in blood: 25 μg/ml) and analyzed by MEA 30 to 120 min following sampling. The first 3 ml of blood were discarded. Blood was taken on day 0 (baseline 1), on day 7 after daily intake of ASA 100 mg for 7 days, on day 7 plus 4 h after the additional intake of 600 mg clopidogrel, after a washout period of 35 days (baseline 2), and on day 35 plus 4 h after 600 mg clopidogrel intake (Figure 1). Each blood sample from all the study participants was exposed to one of the following treatments: saline (spontaneous aggregation), ADP 6.5 μM (ADPtest), cangrelor 100 nM plus ADP 6.5 μM, AA 0.6 mM (ASPItest), cangrelor 100 nM plus AA 0.6 mM, and ASA 5.4 mM (ASA control; standard concentration recommended by the manufacturer) plus AA 0.6 mM.

### Platelet aggregation measurements

Platelet aggregation was measured for 5 min in diluted whole blood by impedance aggregometry using a Multiplate^® ^analyzer, as described previously [19]. Briefly, for the *in vitro *studies a 1:1 mixture of preheated saline and whole hirudin-anticoagulated blood without ASA or with 1 mM ASA was stirred for 3 min at 37°C in single-use Multiplate^®^-test cells. In some experiments, the saline portion of the sample was supplemented with apyrase to inactivate ADP (10 U/ml) or with increasing concentrations of MRS2179 or cangrelor. After the incubation period, platelet aggregation was induced by adding ADP or AA, and measurements were started.

For the *ex vivo *studies, samples of 320 μl of saline (pre-warmed to 37°C) plus 300 μl of hirudin-anticoagulated blood were placed in single-use Multiplate^® ^test cells and stirred for 3 min at 37°C. In some of these experiments, the saline fraction contained cangrelor (100 nM) or ASA (5.4 mM; ASA control). After 3 min of incubation, platelet aggregation was induced by adding either ADP (6.5 μM; ADPtest) or AA (0.6 mM; ASPItest), and measurements were begun. All measurements were performed in duplicate (Figure 1). The area under the curve (AUC) was used to express platelet aggregation in arbitrary aggregation units (AU) multiplied by the recorded time in minutes (AU*min).

### Statistical analysis

Results are given as means ± SD of the number of samples comprising blood of different healthy donors. All platelet function tests were run in duplicate, and means of duplicate determinations were used for figures and statistics. *In vitro *stimulation data were always compared to unstimulated control data by the Mann-Whitney rank sum test or in case of normality and equality of variance by Student's t-test.

Results of *ex vivo *platelet tests obtained after the various treatments were first analyzed for each stimulus for normality by the Kolmogoroff-Smirnoff test and of equality according to Cochran. Then the effect of the various treatments was tested by analysis of variance for overall significance. Post-hoc pair comparisons between treatment groups were then made by the Holm-Sidak-test. For all statistical analyses, a value of p < 0.05 was considered significant.

## Results

### Effect of P2Y_1 _and P2Y_12 _inhibition on ADP-induced aggregation in MEA

**Figure 2:**
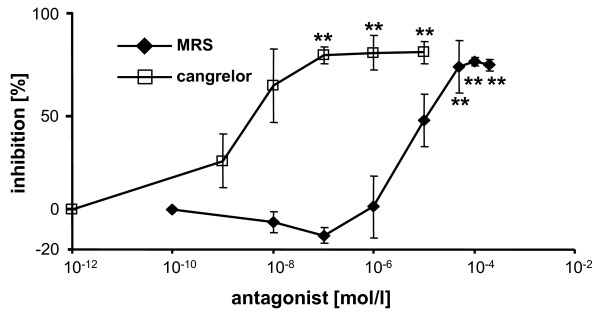
**Inhibition of ADP (5 μM)-induced platelet aggregation by cangrelor (empty squares) and MRS2179 (filled diamonds)**. Data are means ± SD (n = 5). **p < 0.001 for cangrelor/MRS2179 versus control.

Dose-response curves showed concentration-dependent inhibition of ADP-induced platelet aggregation in hirudin-anticoagulated blood by the ADP receptor P2Y_1 _antagonist MRS2179 and by the ADP receptor P2Y_12 _antagonist cangrelor (Figure 2). ADP-stimulated platelet aggregation was inhibited by >90% by maximal concentrations of MRS2179 and cangrelor. Cangrelor (100 nM) reduced ADP (6.5 μM)-induced aggregation of platelets obtained from different blood donors from 530 ± 122 AU*min to 60 ± 33 AU*min (spontaneous aggregation was 57 ± 41 AU*min; n = 5). Inhibition by cangrelor *in vitro *was therefore 99.3 ± 1.4% (p < 0.001). The effective inhibitory concentrations (IC_50_) on platelet aggregation induced by 5 μM ADP were 1.5 μM for MRS2179 and 3 nM for cangrelor.

### Effect of ASA on ADP- and AA- induced platelet aggregation

**Figure 3:**
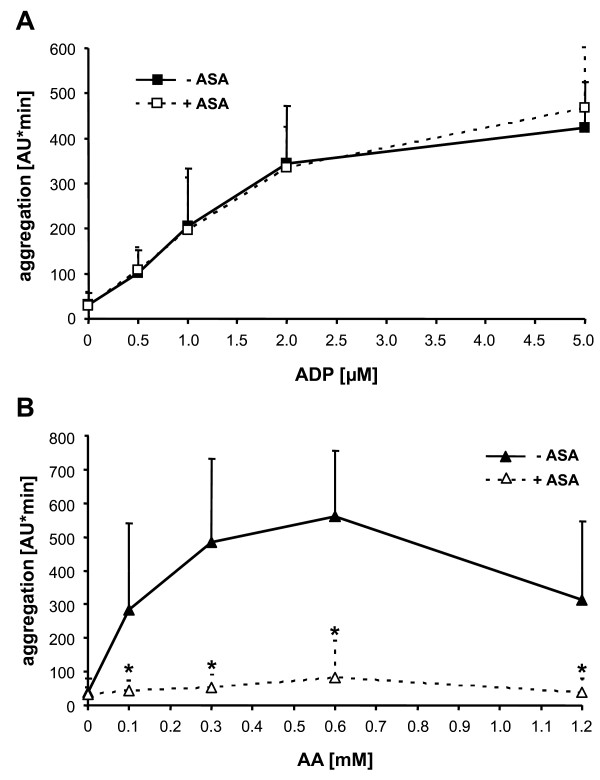
**Effect of ASA (1 mM) on ADP (A)- and AA (B)-induced platelet aggregation**. Data are means ± SD (n = 13). *p < 0.05 for ASA treatment versus control (AA-induced aggregation).

Pre-treatment of blood with ASA (1 mM) had no effect on platelet aggregation stimulated by ADP (0.5-5 μM) (Figure 3A) but significantly reduced AA-stimulated aggregation by > 95% in all individuals tested (n = 13; p < 0.05; Figure 3B). A higher concentration of ASA (5.4 mM) reduced AA (0.6 mM)-induced aggregation of platelets to levels that fell below those of spontaneous aggregation: the respective aggregation values were: for AA 603 ± 83 AU*min; for ASA+AA 18 ± 21 AU*min (spontaneous aggregation 57 ± 41 AU*min; p < 0.001). The two lower concentrations of AA (0.1 and 0.3 mM) did not induce in each individual an aggregation response. Furthermore, for some donors, the aggregation value for the highest AA concentration, 1.2 mM, was lower than that for 0.6 mM AA.

### Effect of apyrase and cangrelor on AA- induced platelet aggregation

**Figure 4:**
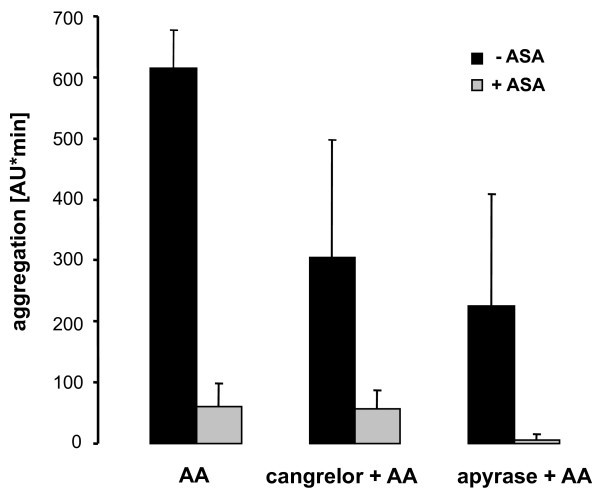
**Effect of the P2Y_12 _antagonist cangrelor (100 nM) and apyrase (10 U/ml) on AA (0.6 mM)-induced platelet aggregation without (control) or with ASA (1 mM)**. Data are means ± SD (n = 3).

AA (0.6 mM)-stimulated platelet aggregation was reduced to a similar degree by cangrelor (100 nM) and by the ADP scavenger enzyme apyrase (10 U/ml), i.e., by 53 ± 26% and 64 ± 29%, respectively (Figure 4). However, pre-treatment of blood with ASA (1 mM) almost completely inhibited AA-mediated platelet aggregation (Figure 4).

Also in the *ex vivo *study (see below), the reduction of AA-stimulated platelet aggregation by cangrelor (100 nM) *in vitro *was assayed. At days 0 and 35 (baselines 1 and 2, respectively), cangrelor reduced AA-induced aggregation from 603 ± 83 AU*min and 636 ± 121 AU*min to 297 ± 121 AU*min and 307 ± 109 AU*min, respectively (n = 5; p < 0.01). This difference amounts to a reduction by cangrelor of AA-induced aggregation of 51% and 52%, respectively.

### Platelet inhibition by ASA, clopidogrel and clopidogrel plus ASA *ex vivo*

**Figure 5:**
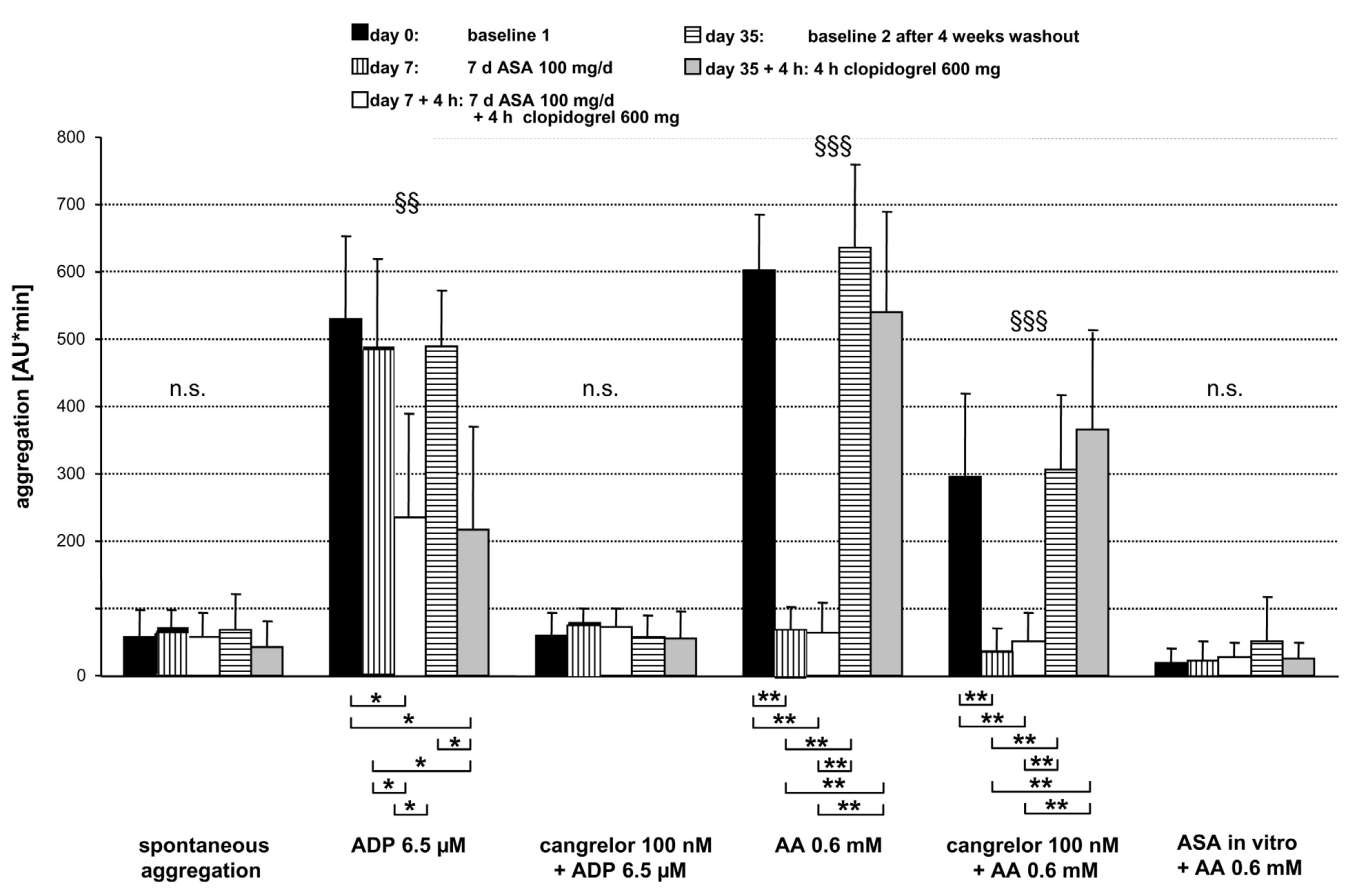
**Platelet inhibition by ASA, clopidogrel and clopidogrel plus ASA *ex vivo***. Platelet aggregation as determined by MEA before drug intake (baseline 1; black bars), after oral intake of ASA (100 mg/d for 7 days; white vertically striped grey bars), ASA (100 mg/d for 7 days) plus clopidogrel (600 mg for 4 h; white bars), after 4 weeks of washout (baseline 2; white horizontally striped dark grey bars), and clopidogrel (600 mg for 4 h; grey bars). Blood from five healthy volunteers was exposed to saline (spontaneous aggregation), ADP (6.5 μM), cangrelor (100 nM) + ADP (6.5 μM), AA (0.6 mM), cangrelor (100 nM) + AA (0.6 mM), ASA *in vitro *(5.4 mM) + AA (0.6 mM). Data are means ± SD (n = 5). The effects of various treatments (after oral intake of ASA, clopidogrel, or both) on platelet aggregation challenged with each stimulus was first tested with overall analysis of variance (n.s. non-significant; §§: p < 0.01; §§§: p < 0.001; on top of the bars) followed by pair comparisons of the individual treatments by the Holm-Sidak-Test, as indicated in brackets under the x-axis (* p < 0.05; ** p < 0.01).

Based on the above *in vitro *results, we performed a feasibility study to evaluate whether MEA can be used to selectively monitor platelet inhibition after oral intake of ASA (100 mg/day for one week) and clopidogrel (a single loading dose of 600 mg) alone and in combination (Figure 1; Methods). Figure 5 summarizes the results of MEA measurements for five healthy individuals before (baseline 1) and after oral intake of the anti-platelet drugs alone or in combination, and after a washout period of 4 weeks (baseline 2). Intra-individual ADP- and AA-stimulated aggregation values did not differ significantly at the two baseline measurements: The mean values of ADP-induced aggregation were 530 ± 123 AU*min and 490 ± 81 AU*min at baselines 1 and 2, respectively; the mean measured values of AA-induced aggregation were 603 ± 83 AU*min and 637 ± 122 at baselines 1 and 2, respectively (means ± SD, n = 5).

Intake of a low dose of ASA for one week resulted in complete inhibition of AA (0.6 mM)-stimulated aggregation in all five individuals (100 ± 3.2%; p < 0.01). Subsequent addition of ASA to the blood samples *in vitro *lowered the aggregation response in some individuals to values that fell below the levels of spontaneous aggregation. ASA intake did not significantly inhibit ADP-induced platelet aggregation in any of the blood donors (Figure 5).

Platelet responsiveness to clopidogrel, determined 4 h after a single 600 mg loading dose, varied in the five volunteers (Figure 5). ADP-induced aggregation was inhibited by <10% in individual 3, and by 100%, 50%, 75% and 85% in individuals 1, 2, 4 and 5, respectively (mean ± SD: 64 ± 35% inhibition; p < 0.05). The *in vitro *addition of cangrelor reduced AA-induced aggregation by 51% (range: 23 to 77%; p < 0.01; see above), whereas ASA intake (alone or in combination with clopidogrel) completely inhibited AA-induced aggregation in all individuals (p < 0.01) (Figure 5).

Clopidogrel intake did not inhibit AA-induced aggregation (Figure 5), except in one volunteer. In this individual, which showed a complete inhibition of ADP-induced aggregation by clopidogrel, AA-stimulated platelet aggregation by clopidogrel was reduced to a degree (by 58%) similar to that seen after *in vitro *incubation with cangrelor (by 65%).

## Discussion

Our results indicate that in MEA ADP-induced aggregation is not influenced by ASA and is therefore specific for ADP receptor blockade (Figures 3 and 5). As shown in Figure 2, ADP-stimulated aggregation was completely inhibited by either P2Y_12 _or P2Y_1 _blockade. The assay presented in this paper is thus P2Y_12 _receptor antagonist selective, on condition that the patient takes drugs that inhibit only the platelet P2Y_12 _receptor (e. g., clopidogrel, prasugrel, or the novel oral reversible P2Y_12 _antagonist AZD6140 [18]) and not the P2Y_1 _receptor. The selectivity of the assay for P2Y_12 _receptor antagonists is based on the findings that: (a) ADP-induced platelet aggregation of hirudin-anticoagulated whole blood is not inhibited by ASA, even at very high concentrations (in contrast to ADP-stimulated platelet aggregation in citrated PRP or suspensions of washed human platelets [18]), and (b) that P2Y_12 _receptor activation is required for ADP-induced aggregation in blood. The maximal inhibitory concentration (100 nM) of the P2Y_12 _receptor antagonist cangrelor completely blocked ADP-mediated aggregation, thereby confirming our previous observations using single-platelet counting [23]. Furthermore, it has previously been shown in patients with acute coronary syndrome that intravenous administration of high concentrations of cangrelor could completely inhibit ADP-induced platelet aggregation, as measured *ex vivo *in blood [24]. It should be noted here that the mechanism of ADP-induced platelet aggregation in whole blood seems to be different from that in PRP and in suspensions of washed human platelets. In PRP and washed platelet suspensions, platelet aggregation is initiated by P2Y_1 _activation and only amplified by the activation of P2Y_12 _[25]. It appears that for ADP-induced platelet aggregation in whole blood there is a strict requirement for concomitant (or sequential) activation of both P2Y receptors and their corresponding signaling pathways.

The most specific assay measuring the function of the clopidogrel target, the P2Y_12 _receptor, is the determination of the state of vasodilator-stimulated phosphoprotein (VASP) phosphorylation by flow cytometry [18,26,27]. This method measures a biochemical parameter, i.e., the decrease of ADP-induced G_i_-mediated dephosphorylation after prior stimulation of G_s_-mediated phosphorylation with prostaglandin E1 (PGE_1_). However, the VASP-based assay - being far more complex than the MEA determination - has some inherent disadvantages: it involves a number of steps (fixation of blood, detergent lysis of platelets) and a number of reagents (PGE_1_, phosphorylation-site specific VASP antibody), and moreover FACS analysis requires sophisticated equipment and an experienced technician. Thus, we conclude that the measurement of the clopidogrel response can be more easily performed by MEA than by measuring VASP phosphorylation.

The bell-shaped dose-response curve of AA-induced platelet aggregation in blood confirms previous studies using washed platelet suspensions [28]. Inhibition of platelet aggregation after exposure to high concentrations of AA is probably due to enhanced formation of platelet inhibitory 12-lipoxygenase metabolites [29].

In the present study, we found that AA-stimulated aggregation of blood was dependent on extracellular ADP and P2Y_12 _receptor activation. The ADP scavenger enzyme apyrase and cangrelor (100 nM) reduced AA-stimulated platelet aggregation to similar degrees. The inhibition of AA-induced aggregation by P2Y_12 _blockade may be explained by the following reasoning: AA-stimulated aggregation of washed platelets and PRP is known to be partially mediated by thromboxane A_2_-induced release of ADP [29]. The situation for AA-stimulated aggregation of platelets in blood might be similar, i.e., thromboxane A_2_-induced secretion of ADP could reinforce platelet aggregation by activation of P2Y_12_.

In all individuals participating in the *ex vivo *study, oral intake of ASA inhibited completely AA-induced aggregation. In contrast, in the small study of healthy volunteers, AA-induced aggregation by oral clopidogrel intake was not significantly reduced. However, we did observe in individual volunteers that after oral clopidogrel intake AA-induced aggregation was reduced when ADP-induced aggregation was completely blocked (data not shown). These observations are in line with our *in vitro *findings of cangrelor on AA-stimulated aggregation (see above). In the light of our results, we predict that in patients taking ASA and treated with potent ADP receptor antagonists such as cangrelor or prasugrel, inhibition of AA-induced aggregation by >95% will indicate that the platelets are also inhibited by ASA. It would be important to validate such an assumption by future studies in a large cohort of cardiovascular patients.

In summary, we have shown (a) that ADP-induced aggregation in MEA is not influenced by ASA both *in vitro *and *ex vivo*; (b) that P2Y_12 _inhibition alone is sufficient to block ADP-induced aggregation in MEA, making this method sufficiently sensitive to monitor the intake of P2Y_12 _receptor antagonists; (c) that a potent P2Y_12 _blockade reduces AA-induced aggregation in MEA both *in vitro *and *ex vivo*; and (d) that ASA inhibited by >95% AA-induced aggregation in MEA *in vitro *and *ex vivo*. We therefore conclude that it is possible to monitor rapidly (5 tests are completed in less than 10 min) and selectively platelet inhibition after intake of ASA and P2Y_12 _antagonists alone or in combination. In view of our promising findings, we suggest that anti-platelet therapy with these two types of anti-platelet drugs can be optimized individually by measuring platelet responsiveness with MEA before and after drug intake.

## Authorship

SP and IB are the principal investigators and take primary responsibility for the paper. They carried out the majority of the experimental work shown in the study and contributed equally to this manuscript. OT performed the first experiments. AC helped in planning the study. WS designed, planned, and coordinated the study. SP, AC and WS wrote the paper. RL did the statistical analysis. All authors read and approved the final manuscript.

## Competing interests

SP is an employee of Dynabyte. AC is co-inventor of the instrument Multiplate^® ^and co-owner of Dynabyte. The other authors have no potential conflicts of interest.
